# Preoperative Extracorporeal Membrane Oxygenation as a Bridge to Cardiac Surgery: Outcomes and Challenges

**DOI:** 10.1016/j.atssr.2025.02.015

**Published:** 2025-03-13

**Authors:** Taylor Pickering, John Eisenga, Cody Dorton, Kyle McCullough, Jasjit Banwait, Jenelle Sheasby, J. Michael DiMaio, Aasim Afzal, Timothy J. George

**Affiliations:** 1 Baylor Scott & White Research Institute, Plano, Texas; 2 Department of Cardiology, Baylor Scott & White The Heart Hospital, Plano, Texas; 3 Department of Cardiothoracic Surgery Baylor Scott & White The Heart Hospital, Plano, Texas

## Abstract

**Background:**

Outcomes of postcardiotomy extracorporeal membrane oxygenation (ECMO) are well studied, but preoperative ECMO bridging is less studied. This single-center review evaluates outcomes of patients supported with ECMO as a bridge to definitive cardiac surgery.

**Methods:**

We retrospectively reviewed all patients who underwent ECMO as a bridge to cardiac surgery between 2013 and 2024. Patients decannulated before surgery or bridged to advanced heart failure therapies were excluded. The primary outcome was survival to hospital discharge. Secondary outcomes included survival to ECMO decannulation, total ECMO duration, and postoperative complications such as hemodialysis, tracheostomy, stroke, reoperation, and survival at 30 days and 1 year.

**Results:**

Sixteen patients were analyzed, of whom 15 (94%) were cannulated for acute heart failure, 1 (6%) for respiratory failure, and 2 (13%) as an adjunct to cardiopulmonary resuscitation. The cohort was 56% female, with a median age of 59.5 years (interquartile range [IQR], 49.3-65.8 years). Surgical procedures included valve surgery (63%), ventricular septal defect repair (31%), and coronary artery bypass grafting (6%). The median ECMO duration was 7 days [IQR, 4-10.5 days]. Survival to decannulation occurred in 81.3%, and 50% survived through hospital discharge. Survivors had lower rates of postoperative dialysis (37.5% vs 87.5%; *P* = .04) but a longer length of stay (25 days vs 9.5 days; *P* = .01).

**Conclusions:**

Preoperative ECMO as a bridge to cardiac surgery is a viable strategy for select high-risk patients, with acceptable survival rates. Larger multicenter studies are needed to refine patient selection and optimize management strategies.

**In Short**

▪ Preoperative ECMO support can stabilize high-risk cardiac patients while bridging to definitive cardiac surgery, thereby achieving a 50% hospital discharge rate.
▪ Survivors demonstrated lower rates of postoperative renal failure requiring dialysis and trended toward shorter ECMO durations.
▪ Multidisciplinary collaboration is critical for patient selection, timing of surgery, and postoperative management.

Extracorporeal membrane oxygenation(ECMO) provides mechanical circulatory or respiratory support for critically ill patients with cardiogenic shock, acute heart failure, or respiratory failure unresponsive to conventional therapies.^1^, ^2^, ^3^ Since its development, ECMO has been increasingly used in adult patients with severe cardiac dysfunction despite its associated risks and complications. In the setting of acute cardiac decompensation, ECMO may provide a period of hemodynamic stability and improvement of multiorgan dysfunction, thus allowing definitive surgical correction under more favorable conditions.^3^, ^4^, ^5^

Although ECMO has shown efficacy as a bridge to recovery or definitive therapies such as transplantation or ventricular assist device implantation, its utility as a bridge to definitive surgical intervention remains understudied. Existing data are mainly small case series and individual reports, with varying outcomes reflecting differing causes, severity of the underlying disease process, and duration of therapy required.^4^^,^^6^, ^7^, ^8^ To address these gaps, we reviewed our institutional experience to better delineate outcomes and inform future patient selection and ECMO use in this context.

## Patients and Methods

We reviewed all patients who underwent venoarterial (VA) ECMO at our center (Baylor Scott & White The Heart Hospital, Plano, TX) between 2013 and 2024 as a bridge to definitive cardiac surgery. Patients decannulated before surgery or those bridged to advanced heart failure therapies, such as left ventricular assist device implantation or transplantation, were excluded from the analysis. Data on demographics, comorbidities, ECMO indications, surgical procedures, and outcomes were collected from electronic medical records. The decision to initiate preoperative VA ECMO support, as well as management of the ECMO circuit, was guided by a multidisciplinary team approach.

The primary outcome of this study was survival to hospital discharge. Secondary outcomes included survival to ECMO decannulation, total ECMO duration, postoperative complications (eg, hemodialysis, tracheostomy, stroke, reoperation), and survival at 30 days and 1 year. Statistical analyses used Mann-Whitney *U* tests and χ^2^ or Fisher exact tests, as appropriate. Predictors of survival to ECMO decannulation and hospital discharge were assessed using univariate linear regression. Kaplan-Meier survival curves were generated to analyze survival trends over time. Statistical significance was defined as *P* <.05. All analyses were performed using R statistical software version 4.4.1 (R Foundation).

This study was Institutional Review Board approved (014-209), with a waiver of informed consent.

## Results

Sixteen patients met inclusion criteria (Table 1). There were no significant baseline differences between survivors and hospital discharge nonsurvivors. Most patients (94%) were cannulated for acute heart failure, whereas 1 (6%) patient was cannulated for respiratory failure. Two patients (13%) required emergency ECMO as an adjunct to cardiopulmonary resuscitation. The index operation included valvular repair or replacement (63%), ventricular septal defect repair (31%), and coronary artery bypass grafting (6%) (Table 2). The median ECMO duration was 7 days (interquartile range [IQR], 4-10.5 days), with a median preoperative duration of 4 days [2-6.3 days]. Survivors trended toward shorter preoperative and overall ECMO durations compared with nonsurvivors (3.5 days [2-4.5 days] vs 5 days [3.3-7.5 days]; *P* = .32; and 4.5 days [3.5-10.5 days] vs 8.5 days [5.8-11 days]; *P* = .43), respectively).

**Table 1:** Baseline Characteristics, Operative Data, and Outcomes of Patients Undergoing Extracorporeal Membrane Oxygenation as a Bridge to Definitive Cardiac Surgery

Characteristics, Operative Data, and Outcomes	All	In-Hospital Mortality	Survival to Discharge	*P* Value
	N = 16	N = 8	N = 8	
Demographics				
Age, y	59.5 [49.3-65.8]	60 [54.5-63.5]	53.5 [44.8-65.8]	.37
Male sex	7 (43.8)	4 (50)	3 (38)	.61
ESRD on HD	2 (12.5)	0 (0)	2 (25)	.45
CAD	9 (56.3)	5 (62.5)	4 (50)	.61
Previous PCI	2 (12.5)	1 (12.5)	1 (12.5)	.57
Previous sternotomy	5 (31.3)	2 (25)	3 (37.5)	.59
Hypertension	10 (62.5)	5 (62.5)	5 (62.5)	1.00
CHF	6 (37.5)	2 (25)	4 (50)	.30
Previous CVA	2 (12.5)	2 (25)	1 (12.5)	.52
Diabetes mellitus	4 (25.0)	3 (37.5)	1 (12.5)	.25
AF	3 (18.8)	2 (25)	1 (12.5)	.52
COPD	2 (12.5)	1 (12.5)	1 (12.5)	1.00
Preoperative and intraoperative				
Days on ECLS, total	7 [4-10.5]	8.5 [5.8-11]	4.5 [3.5-10.5]	.43
Days on ECLS, preoperative	4 [2-6.3]	5 [3.3-7.5]	3.5 [2-4.5]	.32
Days on ECLS, postoperative	1.5 [0-5.5]	1.5 [0-4.8]	2.5 [0-5.5]	1.00
Preoperative Cr, mg/dL	1.2 [0.73-1.8]	1.6 [1.0-2.4]	0.9 [0.6-1.3]	.13
Preoperative EF, %	55 [27.5-55]	37.5 [27.5-58.8]	55 [40-55]	.71
CBP time, min	185 [133-219]	155 [133-255]	186 [142-207]	.87
Cross-clamp time, min	107 [81-138]	104 [62-140]	107 [82-137]	.85
Postoperative outcomes				
Postoperative Cr, mg/dL	1.3 [1.0-1.8]	1.6 [1.3-2.1]	1.0 [0.6-1.3]	**.04**
Postoperative EF, %	45 [25-55]	45 [25-50]	45 [29-55]	.33
RV dysfunction (moderate-severe)	6 (37.5)	4 (50)	2 (25)	.30
RVAD	1 (6.3)	1 (12.5)	0 (0)	1.00
Postoperative HD	10 (62.5)	7 (87.5)	3 (37.5)	**.04**
Tracheostomy	4 (25.0)	0 (0)	4 (50)	.08
Postoperative stroke	1 (6.3)	0 (0)	1 (12.5)	1.00
Reoperation	9 (56.3)	5 (62.5)	4 (50)	.61
LOS, d	18.5 [9.8-25.3]	9.5 [8.5-17.3]	25 [22-34.5]	**.01**

Continuous data are presented as median [interquartile rage], and categorical data are presented as n (%).

The bolded *P*-values represent statistically significant values (ie, *P* < .05).

AF, atrial fibrillation; CAD, coronary artery disease; CBP, cardiopulmonary bypass; CHF, congestive heart failure; COPD, chronic obstructive pulmonary disease; Cr, creatinine; CVA, cerebrovascular accident; ECLS, extracorporeal life support; EF, ejection fraction; ESRD, end-stage renal disease; HD, hemodialysis; LOS, length of stay; PCI, percutaneous coronary intervention; RV, right ventricular; RVAD, right ventricular assist device.

**Table 2:** Clinical Course and Outcomes of Patients Supported With Extracorporeal Membrane Oxygenation as a Bridge to Cardiac Surgery

Patient	Age, y	Sex	ECLS Type and Indication	Surgery After ECMO	ECMO Duration (Preoperative, Postoperative, Overall), d	Outcome
1	27	M	VA ECMO: thrombosed mitral valve replacement	Third MVR	6, 10, 16	1-y survival
2	62	M	VA ECMO: acute RCA occlusion after PCI, postinfarct VSD	CABG, MVr, VSD repair	7, 7, 14	In-hospital mortality
3	78	F	VA ECMO: severe AS, after AVR/CABG with instability	Emergency redo AVR, TVr	1 ,0, 1	Death before decannulation
4	47	M	VA ECMO: severe HF after MVR/TVR with prosthetic endocarditis	Redo MVR	3, 7, 10	1-y survival
5	59	F	VA ECMO: acute MR after balloon valvuloplasty	MVR	5, 1, 6	Death before decannulation
6	68	F	VA ECMO: acute respiratory failure/shock after AVR	Third AVR	4, 0, 4	1-y survival
7	68	F	VA ECMO: cardiogenic shock after large anterior MI, VSD	CABG, VSD repair	4, 4, 8	In-hospital mortality
8	53	M	VA ECMO: cardiogenic shock after large anterior MI, VSD	VSD repair, Impella ventricular assist device (Abiomed) repositioning	5, 0, 5	In-hospital mortality
9	41	F	VA ECMO: severe mitral stenosis, acute on chronic CHF	Septal myomectomy, MVR	2, 0, 2	1-y survival
10	61	F	VA/VV ECMO: ECPR 2/2 STEMI, cardiac arrest, LAD dissection	Emergency off-pump CABG	0, 10, 10	Death before decannulation
11	65	F	VA ECMO: septal myomectomy with LV offloading post-VSD	Redo sternotomy, VSD repair	0, 5, 5	1-y survival
12	68	F	VA ECMO: ECPR, severe AS and CHF	AVR, MVr	4, 0, 4	1-y survival
13	55	M	VA ECMO: cardiogenic shock after large anterior MI, VSD	LV rupture repair, VSD repair	9, 0, 9	In-hospital mortality
14	60	M	VA ECMO: cardiogenic shock after large anterior MI, VSD	VSD repair	7, 5, 12	1-y mortality
15	46	F	VA ECMO: mixed cardiogenic-septic shock from endocarditis	MVR, TVR	2, 0, 2	1-y survival
16	50	M	LAVA ECMO: VT storm after VA LAVA ECMO	Redo sternotomy, MVR, RVAD	15, 2, 17	In-hospital mortality

AS, aortic stenosis; AVR, aortic valve replacement; CABG, coronary artery bypass grafting; CHF, congestive heart failure; ECLS, extracorporeal life support; ECMO, extracorporeal membrane oxygenation; ECPR, extracorporeal cardiopulmonary resuscitation; F, female; HF, heart failure; LAD, left anterior descending artery; LAVA, left atrial venoarterial; LV, left ventricular; M, male; MI, myocardial infarction; MR, mitral regurgitation; MVr, mitral valve repair; MVR, mitral valve replacement; PCI, percutaneous coronary intervention; RCA, right coronary artery; RVAD, right ventricular assist device; STEMI, ST-segment elevation myocardial infarction; TVr, tricuspid valve repair; TVR, tricuspid valve replacement; VA, venoarterial; VSD, ventricular septal defect; VT, ventricular tachycardia; VV, venovenous.

Survival to ECMO decannulation and hospital discharge was 81.3% and 50%, respectively. Of the 8 in-hospital deaths, most occurred early in the postoperative period. Kaplan-Meier analysis demonstrated that 30-day mortality and 1-year mortality were similar (50% vs 56.3%) (Supplemental Figure 1). The median follow-up for survivors was 45 days [7-928 days]. Univariable analysis revealed that neither demographic characteristics nor intraoperative factors were predictive of survival to decannulation or discharge (Supplemental Table, Supplemental Figure 2). Survivors had significantly lower postoperative creatinine levels (1.0 mg/dL [0.6-1.3 mg/dL] vs 1.6 mg/dL [1.3-2.1 mg/dL]; *P* = .04) and decreased dialysis requirements (37.5% vs 87.5%; *P* = .04). Reoperation for bleeding was required in 50% of patients, and prolonged ventilation necessitated tracheostomy in 25%. The median length of stay was 25 days for survivors vs 9.5 days for nonsurvivors (*P* = .01).

## Comment

In this cohort, 50% of patients supported with VA ECMO as a bridge to cardiac surgery survived to discharge, a finding that aligns with rates reported by other series.^4^, ^5^, ^6^, ^7^, ^8^ Outcomes vary widely, however, a reflection of small cohort sizes, differences in underlying causes, variable patient selection, institutional practices, and EMCO protocols. Unique to this cohort is the disproportionate number of postinfarction ventricular septal defects, a subgroup with a notably increased risk of mortality, which likely adversely affected our survival outcomes.^4^^,^^7^ Mortality stabilized after approximately 2 months, thus suggesting that early perioperative complications largely drive outcomes in this high-risk population (Supplemental Figure 1).^6^, ^7^, ^8^

Given our limited sample size, logistic regression did not reveal definitive predictors of survival. However, patients who died demonstrated higher rates of postoperative renal failure requiring dialysis and a trend toward longer ECMO durations, factors previously associated with worse outcomes and greater disease severity.^3^^,^^8^ Rastan and collelagues^5^ emphasized that extended ECMO support correlated with increased mortality and morbidity, including bleeding, infections, and multiorgan dysfunction, which may further worsen the patient’s clinical status. Although renal replacement therapy (RRT) is commonly required in ECMO-treated patients, its use is associated with an 89% higher mortality rate than in patients not requiring RRT.^9^ However, studies that adjusted for disease severity and implemented early RRT demonstrated a trend toward reduced mortality rates, a finding suggesting that timely intervention may mitigate some of the adverse outcomes associated with acute kidney injury.

Finally, the importance of a multidisciplinary heart team approach, involving cardiac surgeons, intensivists, and cardiologists, remains crucial in managing these complex patients. In our practice, the duration of preoperative ECMO support was guided by ongoing assessments of each patient’s hemodynamic stability, degree of mechanical circulatory support required, normalization of end-organ function (particularly renal function), euvolemia, and the severity of the underlying disease, all balanced against the potential risks of prolonged support. Frequent reevaluation and open communication between the operative surgeon and other members of the care team were critical given the lability of each patient’s clinical status.^10^ Further investigations aimed at determining the optimal ECMO initiation and surgical intervention timing, refining patient selection, and optimizing RRT initiation thresholds, fluid management strategies, and adjunctive therapies to reduce acute kidney injury in this vulnerable population are needed to improve overall outcomes.

This study has several notable limitations. Given its small sample size, single-center design, and retrospective design, statical power and generalizability are limited. Additionally, the lack of standardized protocols for anticoagulation, renal support, and ECMO weaning at our institution may also introduce variability in outcomes.

In conclusion, preoperative ECMO as a bridge to cardiac surgery offers a lifesaving option for select high-risk patients, with reasonable survival rates. Prolonged ECMO duration and postoperative renal dysfunction suggested greater disease severity and portend worse outcomes, thus highlighting the importance of careful patient selection and the need for strategies to optimize timing and reduce complications. Finally, a multidisciplinary team approach remains essential for successful outcomes.
