# Supplementary material for: Preoperative Extracorporeal Membrane Oxygenation as a Bridge to Cardiac Surgery: Outcomes and Challenges
Source: Ann Thorac Surg Short Rep. 2025 Mar 13;3(3):772–6. doi: 10.1016/j.atssr.2025.02.015 (PMC12559571; doi:10.1016/j.atssr.2025.02.015)

**Supplementary File**

| **Supplemental Table.** Predictors of Survival to Hospital Discharge and Decannulation in Patients Undergoing ECMO as a Bridge to Cardiac Surgery | | | | | | | | | |
| --- | --- | --- | --- | --- | --- | --- | --- | --- | --- |
|  | **Survival to Hospital Discharge** | | | |  | **Survival to Decannulation** | | | |
|  | **OR** | **95% Lower CI** | **95% Upper CI** | **p-value** |  | **OR** | **95% Lower CI** | **95% Upper CI** | **p-value** |
| **Demographics** |  |  |  |  |  |  |  |  |  |
| Age | 1.06 | 0.96 | 1.17 | 0.22 |  | 1.12 | 0.95 | 1.33 | 0.18 |
| Sex | 1.67 | 0.23 | 12.22 | 0.62 |  | 0.00 | 0.00 | Inf | 1.00 |
| ESRD on HD | 0.00 | 0.00 | Inf | 0.99 |  | 0.00 | 0.00 | Inf | 1.00 |
| CAD | 1.67 | 0.23 | 12.22 | 0.62 |  | 1.71 | 0.12 | 23.94 | 0.69 |
| Prior PCI | 1.00 | 0.05 | 19.36 | 1.00 |  | 0.00 | 0.00 | Inf | 1.00 |
| Prior Sternotomy | 0.56 | 0.06 | 4.76 | 0.59 |  | 1.13 | 0.08 | 16.31 | 0.93 |
| HTN | 1.00 | 0.13 | 7.57 | 1.00 |  | >100 | 0.00 | Inf | 1.00 |
| CHF | 0.33 | 0.04 | 2.77 | 0.31 |  | 0.80 | 0.06 | 11.30 | 0.87 |
| Previous CVA | 1.00 | 0.05 | 19.36 | 1.00 |  | 0.00 | 0.00 | Inf | 1.00 |
| DM | 4.20 | 0.33 | 53.12 | 0.27 |  | 1.67 | 0.11 | 25.43 | 0.71 |
| Afib | 2.33 | 0.17 | 32.58 | 0.53 |  | 2.75 | 0.16 | 46.79 | 0.48 |
| COPD | 1.00 | 0.05 | 19.36 | 1.00 |  | 6.00 | 0.26 | 140.04 | 0.26 |
| **Pre- and intra-operative** |  |  |  |  |  |  |  |  |  |
| Days on ECLS | 1.01 | 0.81 | 1.25 | 0.96 |  | 0.83 | 0.60 | 1.16 | 0.28 |
| Days on ECLS Preop | 1.20 | 0.86 | 1.67 | 0.28 |  | 0.56 | 0.25 | 1.24 | 0.15 |
| Days on ECLS Postop | 0.98 | 0.73 | 1.31 | 0.88 |  | 0.94 | 0.63 | 1.38 | 0.74 |
| Preop Cr | 0.94 | 0.67 | 1.31 | 0.71 |  | 0.81 | 0.31 | 2.08 | 0.66 |
| Preop Ef | 0.99 | 0.94 | 1.04 | 0.72 |  | 1.01 | 0.95 | 1.08 | 0.66 |
| CBP Time | 1.01 | 0.99 | 1.02 | 0.57 |  | 1.01 | 0.98 | 1.04 | 0.45 |
| Clamp Time | 0.99 | 0.97 | 1.02 | 0.70 |  | 0.99 | 0.93 | 1.04 | 0.62 |
| **Postoperative Outcomes** |  |  |  |  |  |  |  |  |  |
| Postop Cr | 3.42 | 0.63 | 18.44 | 0.15 |  | 0.91 | 0.19 | 4.22 | 0.90 |
| Postop EF | 0.97 | 0.91 | 1.03 | 0.30 |  | 0.96 | 0.88 | 1.06 | 0.45 |
| RV Dysfunction |  |  |  |  |  |  |  |  |  |
| None | Reference |  |  |  |  |  |  |  |  |
| Mild | 2.00 | 0.15 | 26.73 | 0.60 |  | 0.67 | 0.04 | 11.29 | 0.78 |
| Moderate | >100 | 0.00 | Inf | 1.00 |  | 0.00 | 0.00 | Inf | 1.00 |
| Severe | 3.00 | 0.25 | 35.33 | 0.38 |  | 0.00 | 0.00 | Inf | 1.00 |
| RVAD | >100 | 0.00 | Inf | 1.00 |  | 0.00 | 0.00 | Inf | 1.00 |
| HD postop | 11.67 | 0.92 | 147.56 | 0.06 |  | 1.25 | 0.09 | 17.65 | 0.87 |
| Trach | 0.00 | 0.00 | Inf | 1.00 |  | 0.00 | 0.00 | Inf | 1.00 |
| Post op stroke | 0.00 | 0.00 | Inf | 1.00 |  | 0.00 | 0.00 | Inf | 1.00 |
| Reoperation | 1.67 | 0.23 | 12.22 | 0.62 |  | 1.71 | 0.12 | 23.94 | 0.69 |
| LOS | 0.79 | 0.62 | 0.99 | 0.04 |  | 0.00 | 0.00 | Inf | 1.00 |
| *Odds ratios (OR), 95% confidence intervals (CI), and p-values are presented for predictors of survival to hospital discharge and survival to decannulation. ESRD, end-stage renal disease; HD, hemodialysis; CAD, coronary artery disease; PCI, percutaneous coronary intervention; HTN, hypertension; CHF, congestive heart failure; CVA, cerebrovascular accident; DM, diabetes mellitus; Afib, atrial fibrillation; COPD, chronic obstructive pulmonary disease; ECLS, extracorporeal life support; Cr, creatinine; EF, ejection fraction; CBP, cardiopulmonary bypass; RVAD, right ventricular assist device; LOS, length of stay. "Inf" denotes infinite confidence intervals due to limited event counts or extreme effect sizes.* | | | | | | | | | |

**Supplementary Figure 1.** Kaplan-Meier survival curve demonstrating the survival probability for patients placed on pre-operative ECMO as a bridge to cardiac surgery.

**
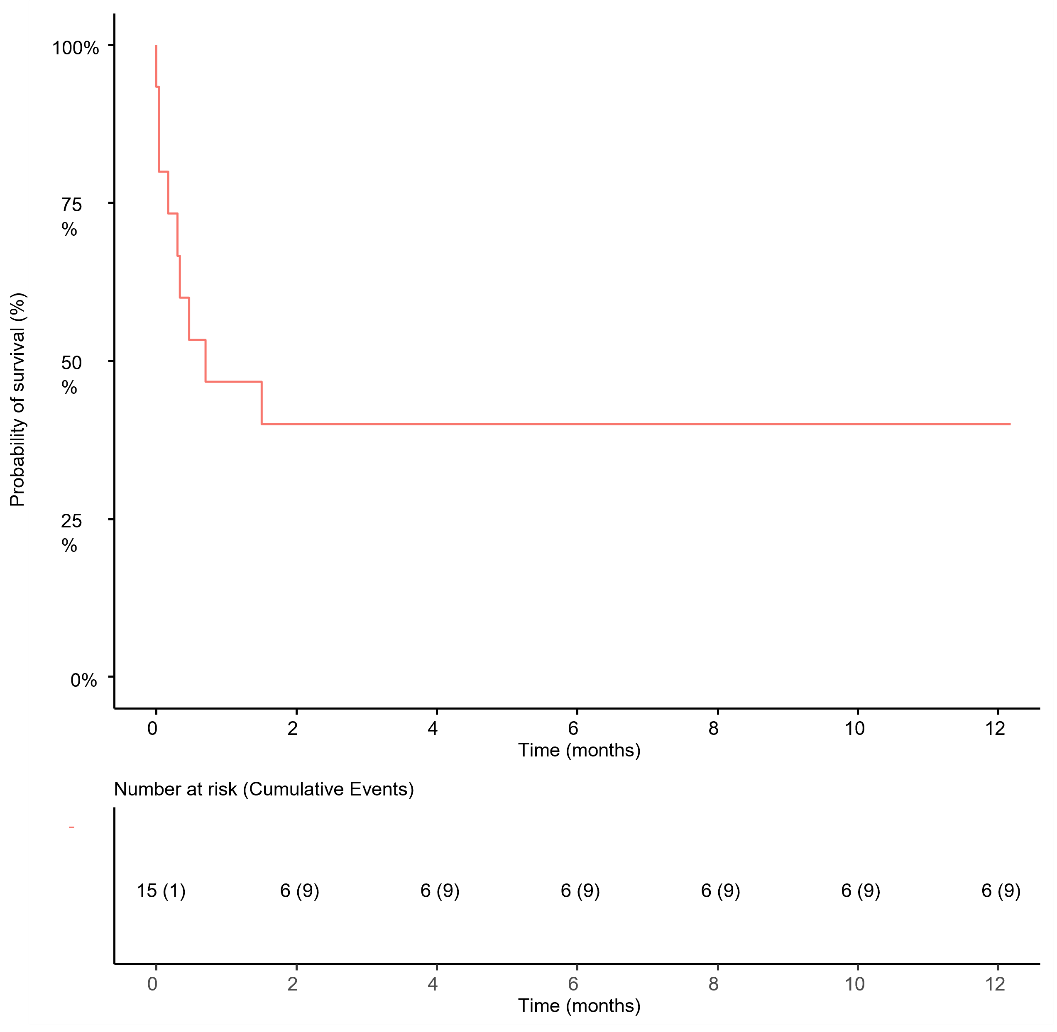
**

**Supplementary Figure 2a and b**: Odds ratios with 95% confidence intervals for predictors of (a) survival to ECMO decannulation and (b) survival to hospital discharge, based on logistic regression from Supplementary Table 1.


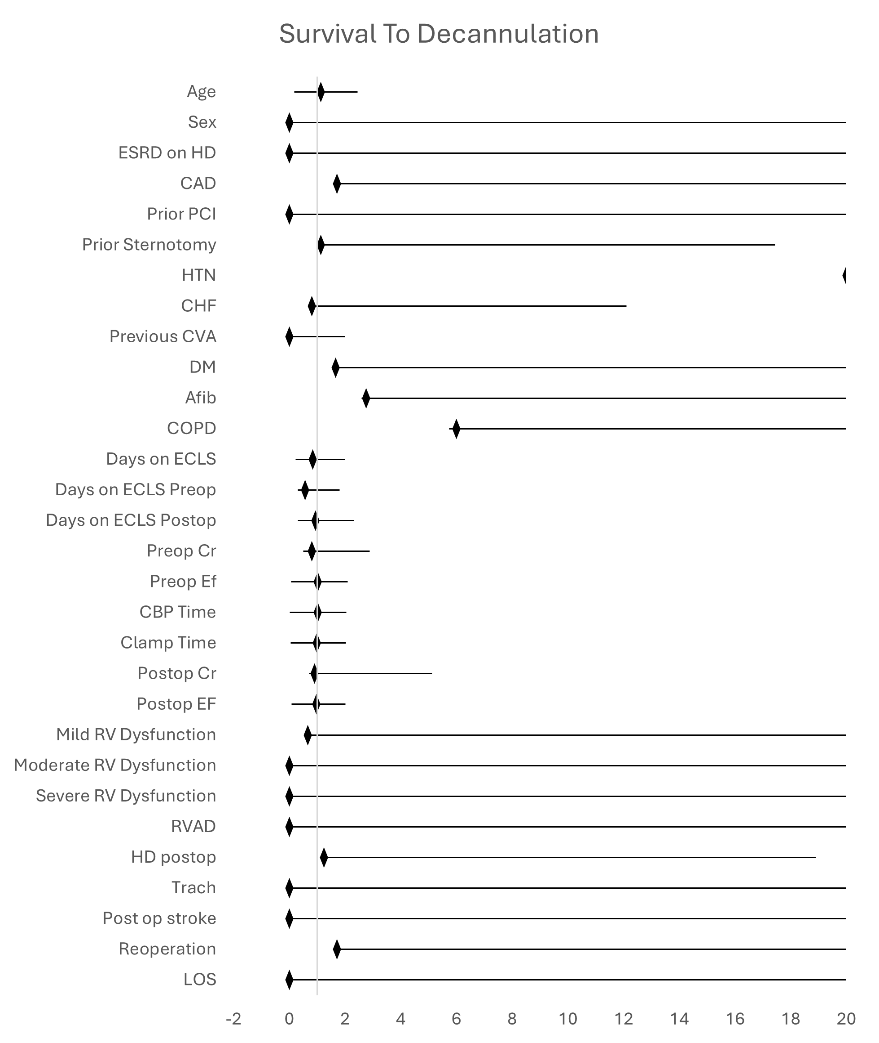

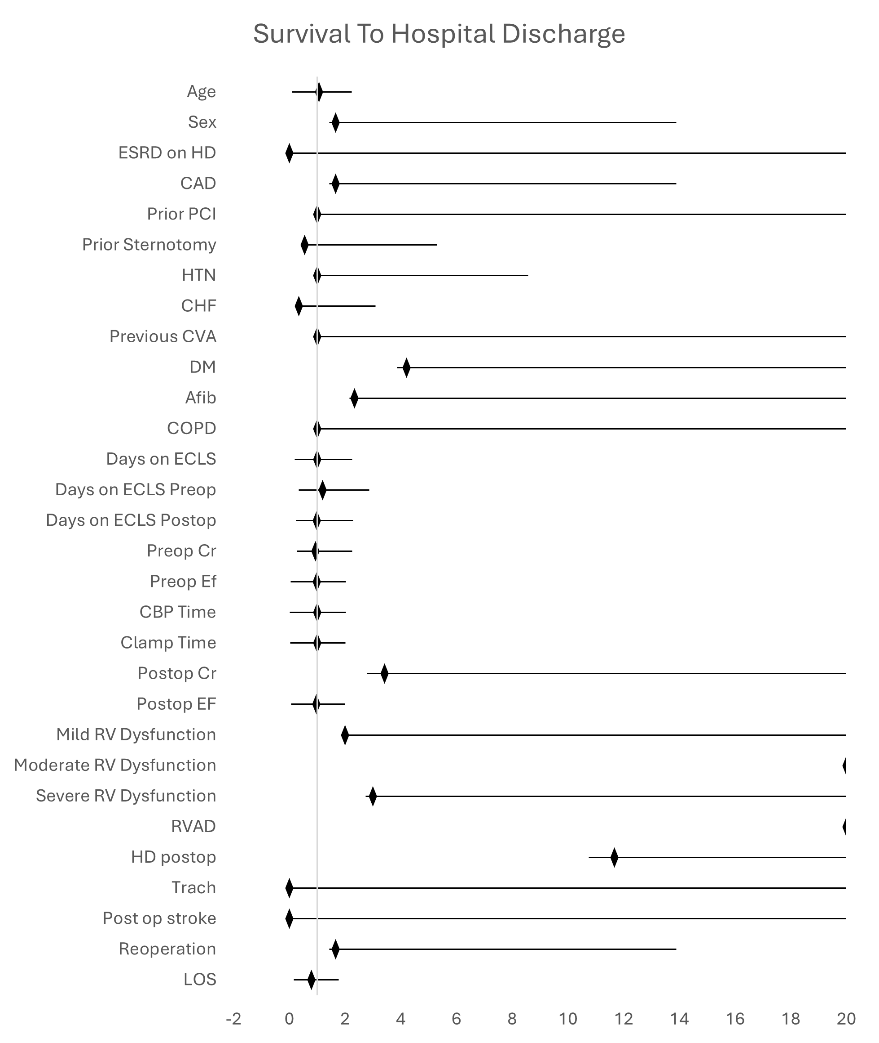

Supplement: Supplementary Table 1 and Supplementary figures 1-2 [file mmc1.docx]
